# Relationship Between Changes in Serum Levels of Intact Parathyroid Hormone and Sclerostin After a Single Dose of Zoledronic Acid: Results of a Phase 1 Pharmacokinetic Study

**DOI:** 10.1007/s00223-021-00900-w

**Published:** 2021-08-24

**Authors:** Tatsuhiko Kuroda, Masataka Shiraki, Toshitaka Nakamura, Hiroaki Suzuki, Kazuki Hiraishi, Toshitsugu Sugimoto, Satoshi Tanaka

**Affiliations:** 1 Healthcare R&D Center, Asahi Kasei Corporation, 1-1-2 Yurakucho, Chiyoda-ku, Tokyo, 100-0006 Japan; 2 Research Institute and Practice for Involutional Diseases, 1610-1 Meisei, Misato, Azumino, Nagano 399-8101 Japan; 3 Touto Sangenjaya Rehabilitation Hospital, 1-24-3 Sangenjaya, Setagaya-ku, Tokyo, 154-0024 Japan; 4 Medical Affairs Department, Asahi Kasei Pharma Corporation, 1-1-2 Yurakucho, Chiyoda-ku, Tokyo, 100-0006 Japan; 5 Internal Medicine 1, Faculty of Medicine, Shimane University, 89-1 Enya-cho, Izumo, Shimane 693-8501 Japan; 6 Development Planning, Clinical Development Center, Asahi Kasei Pharma Corporation, 1-1-2 Yurakucho, Chiyoda-ku, Tokyo, 100-0006 Japan

**Keywords:** Zoledronic acid, Sclerostin, Parathyroid hormone, Bone formation

## Abstract

Although changes in serum sclerostin levels at 12 months after infusion of zoledronic acid have been reported, the changes in sclerostin levels at earlier time points are poorly understood. We reanalyzed the study data of a previous phase 1 pharmacokinetic study and investigated the correlation between changes in sclerostin levels and relevant factors in calcium metabolism. A total of 24 Japanese female subjects with primary postmenopausal osteoporosis were administered a single 4- or 5-mg dose of zoledronic acid. Serum and urine samples were collected on days 15, 29, 90, 180, and 365 after administration. Serum levels of calcium, phosphate, intact parathyroid hormone (iPTH), and sclerostin were measured. Levels of serum sclerostin were unchanged from baseline on days 15 and 29, but increased significantly on day 90, subsequently decreased significantly on day 180, and returned to baseline levels on day 365. A significant negative correlation was observed between changes in iPTH levels at early time points and sclerostin levels at later time points. This suggests that sclerostin was negatively regulated by iPTH, and the decrease in sclerostin may indicate the start of bone formation during later time points after zoledronic acid injection.

**Supplementary Information:**

The online version contains supplementary material available at 10.1007/s00223-021-00900-w.

## Introduction

Zoledronic acid is categorized as a nitrogen-containing bisphosphonate (BP) with an antiosteoclastic effect [1]. Once-yearly injection of zoledronic acid 5 mg has been widely used for the treatment of osteoporosis [2]. Zoledronic acid significantly increases bone mineral density and reduces fracture risk at vertebral and nonvertebral sites [3, 4]. Several studies have shown rapid decreases in bone resorption markers and subsequent decreases in bone formation markers after zoledronic acid infusion as a result of its antiosteoclastic effect [4, 5].

Recently, there has been accumulating evidence that osteocyte-derived proteins play a role in the regulation of bone formation [6]. In particular, sclerostin, a product of the *SOST* gene, has attracted attention, as it negatively regulates Wnt signaling and bone formation [7, 8]. Several clinical trials have demonstrated differences in the expression of sclerostin depending on the agents used in osteoporosis treatment [9–12].

Furthermore, several studies have reported changes in serum sclerostin levels following infusion of zoledronic acid [13–16]. Previous studies have shown that sclerostin levels were not significantly changed from baseline at 12 months after zoledronic acid infusion. However, limited data are available on the changes in sclerostin levels prior to the 12-month time point. We therefore aimed to investigate the changes in sclerostin levels over 12 months after infusion of zoledronic acid using more frequent time points.

In this study, we measured serum sclerostin levels and other bone turnover markers at more frequent time points than those used in our previous phase 1 pharmacokinetic (PK) study [17] and performed a subanalysis to investigate the correlation between changes in sclerostin levels and relevant factors in calcium metabolism to determine how zoledronic acid affects bone formation.

## Materials and Methods

### Study Subjects and Treatment

This post hoc analysis was conducted using data from the PK study, which was a single-administration study with a single-blind design [17]. Twenty-four Japanese female subjects (age: 45–79 years) diagnosed as having primary postmenopausal osteoporosis using the criteria for primary osteoporosis of the Japanese Society for Bone and Mineral Research were enrolled [18]. A single 4- or 5-mg dose of zoledronic acid was administered intravenously over a period of at least 15 min. Subjects with a history of BP use within the 2-year period before the study, kidney disease, abnormal serum calcium levels, or history of diabetes mellitus with diabetic complications were excluded.

Serum and urine samples were collected on days 15, 29, 90, 180, and 365 after drug administration. Oral supplemental calcium (460 mg) and vitamin D (10.0 μg) were administered daily during the study period.

### Measured Parameters

Serum levels of calcium, phosphate, and intact parathyroid hormone (iPTH) were measured. iPTH was measured using the Elecsys PTH assay (Roche Diagnostics K.K., Tokyo, Japan; inter-assay coefficient of variation: 2.3–2.5%). Serum levels of sclerostin were evaluated using an enzyme-linked immunosorbent assay (ELISA) kit (SCLEROSTIN; Biomedica Medizinprodukte, GmbH & Co KG, Vienna, Austria; inter-assay coefficient of variation: 2.5–7.5%). All samples were analyzed by Mitsubishi Chemical Medience (Tokyo, Japan).

This study was conducted in compliance with the Declaration of Helsinki and Good Clinical Practice guidelines. The study protocol was reviewed by the institutional review board of the study centers. Written informed consent was obtained from all subjects before administration of zoledronic acid.

### Statistical Analysis

Measured parameters are summarized as mean ± standard deviation (SD). Baseline differences in calcium metabolism and sclerostin between the 4- and 5-mg doses were evaluated using analysis of variance (ANOVA). Percent changes from baseline were evaluated using a paired *t* test. Differences in percent changes between the 4- and 5-mg doses were evaluated by ANOVA. The relationship between calcium metabolism and sclerostin was evaluated using Spearman’s correlation method for the whole set of data and excluding outliers. *p*-values < 0.05 were considered statistically significant.

## Results

**Fig. 1:**
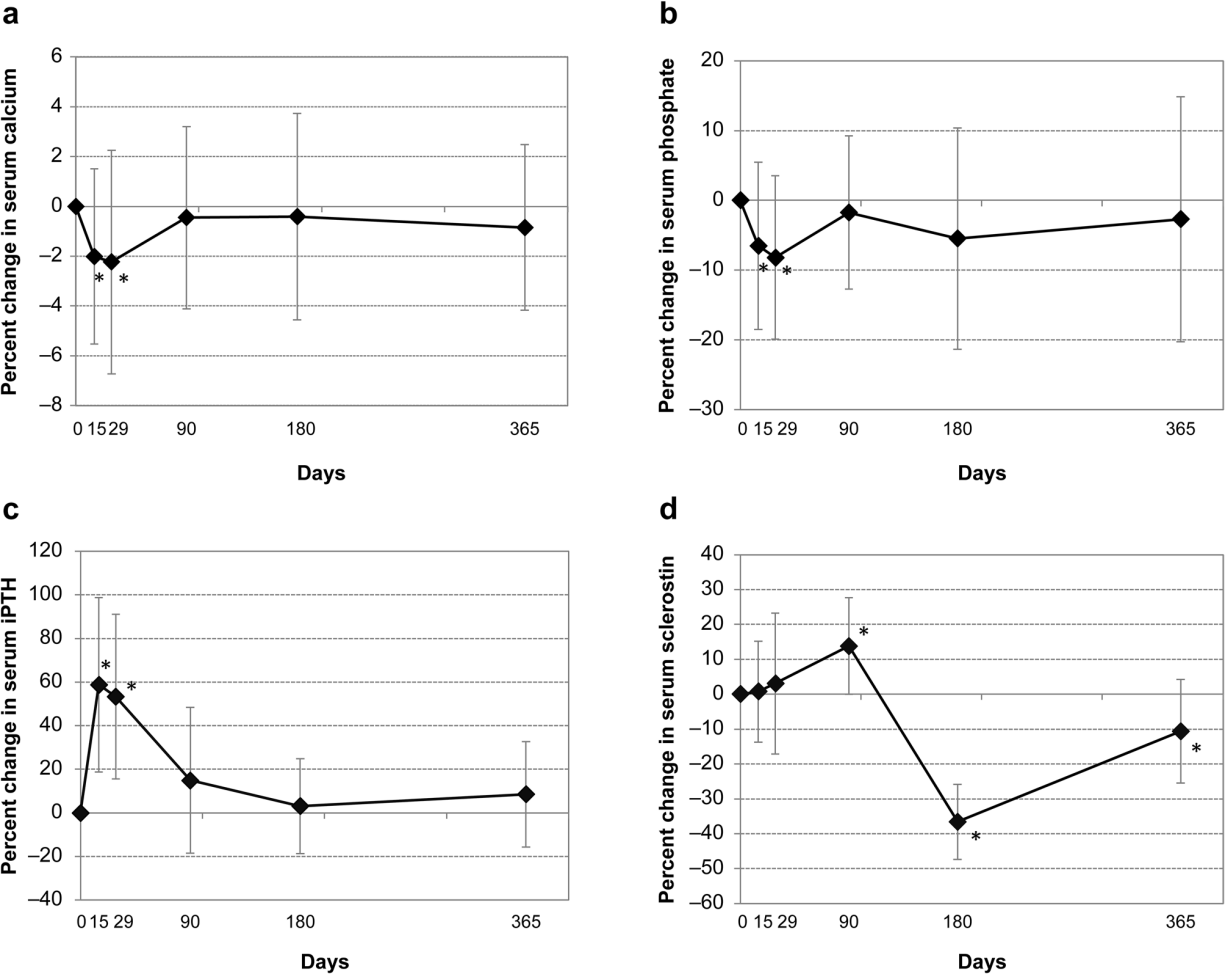
Time-dependent percent changes in **a** serum calcium, **b** serum phosphate, **c** serum iPTH, and **d** serum sclerostin. Data are mean percent change ± standard deviation. *p < 0.05 versus baseline. *iPTH* intact parathyroid hormone

Twenty-four subjects were randomized to the zoledronic acid 4-mg (*n* = 12) or 5-mg (*n* = 12) group. No significant differences were observed in baseline characteristics such as age, body mass index, or years after menopause between the groups [17]. The mean ± SD age in the 4- and 5-mg groups was 66.8 ± 5.7 and 66.2 ± 8.2 years, respectively. Baseline levels of serum calcium, phosphate, iPTH, and sclerostin are shown in Table 1. There were no significant differences between the 4- and 5-mg groups. Percent changes in serum calcium, phosphate, and iPTH (integrating data from the 4- and 5-mg groups) are shown in Fig. 1a–c.

**Table 1:** Baseline characteristics

	Zoledronic acid 4-mg group	Zoledronic acid 5-mg group	*p*-value
Serum calcium (mg/dL)	9.1 ± 0.4	9.2 ± 0.4	0.642
Serum phosphate (mg/dL)	3.7 ± 0.5	3.6 ± 0.4	0.402
Serum iPTH (pg/mL)	39.6 ± 11.6	49.5 ± 14.0	0.072
Serum sclerostin (pmol/L)	973.4 ± 316.5	949.6 ± 286.4	0.848

Data are mean ± SD

*iPTH* intact parathyroid hormone, *SD* standard deviation

After administration of zoledronic acid, rapid decreases were observed in serum calcium and phosphate on days 15 and 29 in both groups. At the same time points, serum iPTH increased reciprocally. Levels of serum sclerostin were unchanged from baseline on days 15 and 29; however, they increased significantly on day 90, subsequently decreased significantly on day 180, and returned to baseline levels on day 365 (Fig. 1d).

Correlations between the percent changes in the parameters from baseline to each measurement time point were investigated. Correlations of the changes in levels of calcium as well as phosphate and iPTH at earlier time points are shown in Online Resource 1 using the whole set of data. One outlier each was observed for iPTH at days 0–15 and days 0–29. Table 2 summarizes the results of the reanalysis excluding the outliers. The correlations and statistical differences in the parameters were similar between the whole set of data and excluding the outliers. Increases in iPTH on days 15 and 29 were significantly correlated with decreases in serum calcium on day 15 and/or day 29, and the increase in iPTH on day 15 was significantly correlated with a decrease in serum phosphate on day 15 (Table 2).

**Table 2:** Correlation of changes in calcium and phosphate with percent changes in iPTH excluding outliers

	iPTH (days 0–15)		iPTH (days 0–29)	
	*R*	*p*-value	*R*	*p*-value
Serum calcium				
Days 0–15	– 0.628	0.001	– 0.446	0.033
Days 0–29	– 0.358	0.093	– 0.450	0.031
Serum phosphate				
Days 0–15	– 0.513	0.012	– 0.128	0.560
Days 0–29	– 0.096	0.663	– 0.228	0.295

*iPTH* intact parathyroid hormone

**Fig. 2:**
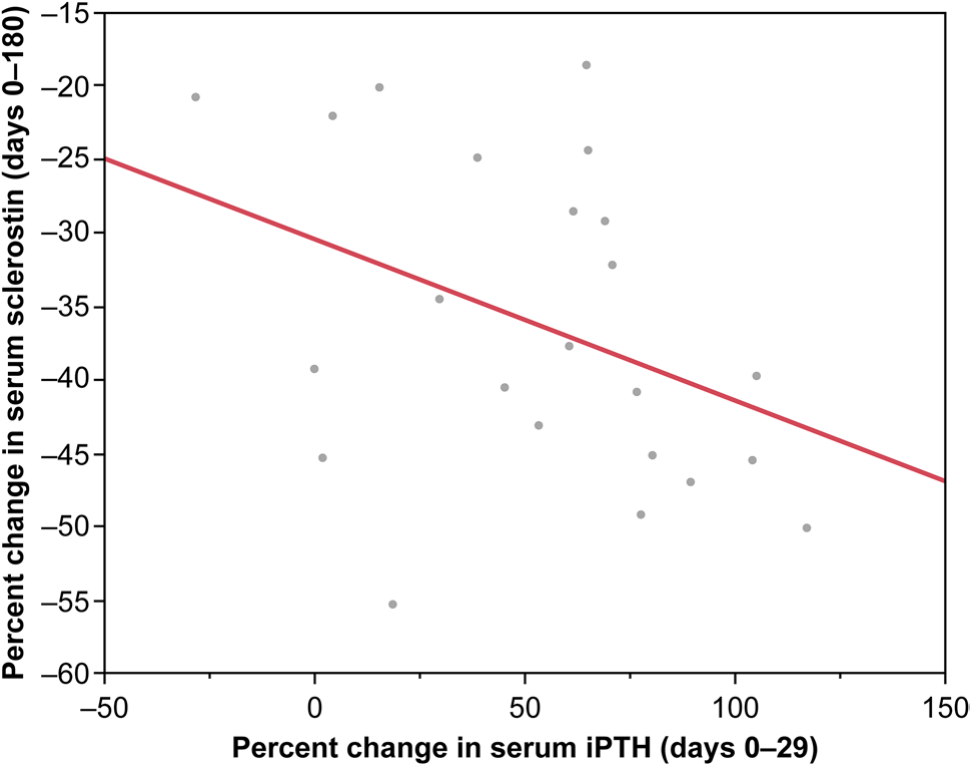
Correlation between percent change in serum iPTH and sclerostin excluding outliers. *iPTH* intact parathyroid hormone

Online Resource 2 shows the correlations of percent changes in levels of calcium, phosphate, and iPTH at earlier time points (days 15 and 29) with percent changes in levels of serum sclerostin on days 90 and 180 using the whole set of data. One outlier each was observed for iPTH at days 0–15 and days 0–29. Table 3 summarizes the results of the reanalysis excluding the outliers. The correlations and statistical differences in the parameters were similar between the whole set of data and excluding the outliers. Online Resource 3 shows the correlations of percent changes in levels of iPTH at days 0–29 with percent changes in levels of sclerostin at days 0–180 using the whole set of data. Figure 2 shows the correlation between percent changes in levels of iPTH at days 0–29 and sclerostin at days 0–180 excluding the outliers. Increases in iPTH on day 29 significantly contributed to the subsequent decrease in serum sclerostin (day 180; *R*^2^ = 0.186; Fig. 2). The relationship and trend were similar between the whole set of data and excluding the outliers.

**Table 3:** Correlation of percent changes in iPTH, calcium, and phosphate with percent changes in sclerostin excluding outliers

	Serum sclerostin (days 0–90)		Serum sclerostin (days 0–180)	
	*R*	*p*-value	*R*	*p*-value
iPTH				
Days 0–15	– 0.075	0.733	– 0.193	0.377
Days 0–29	– 0.075	0.733	– 0.431	0.040
Serum calcium				
Days 0–15	– 0.013	0.952	0.260	0.220
Days 0–29	– 0.157	0.465	0.269	0.203
Serum phosphate				
Days 0–15	0.059	0.785	0.045	0.834
Days 0–29	– 0.039	0.856	0.318	0.130

*iPTH* intact parathyroid hormone

## Discussion

In this analysis, decreases in serum calcium and phosphate were correlated with increases in iPTH at earlier time points after the injection of zoledronic acid. Moreover, increases in iPTH at earlier time points contributed to subsequent decreases in sclerostin.

Changes in sclerostin levels after administration of BPs have been reported previously [12–14, 19, 20]. However, the changes were not significant at 19 months after oral alendronate or risedronate treatment [12]. On the other hand, ibandronate increased the serum level of sclerostin at 1 month and maintained the levels above baseline until 18 months [19, 20]. Several studies have reported on the transition of serum sclerostin levels after administration of zoledronic acid. Catalano et al. reported that serum sclerostin levels peaked on day 7, with a significant increase observed at 30 days, before returning to baseline beyond 360 days after administration of zoledronic acid [13]. On the other hand, Anastasilakis et al. reported that serum sclerostin levels significantly decreased at 3 months after zoledronic acid administration [14]. These reports indicate that BPs result in sclerostin increases at earlier time points after injection and subsequently result in decreases at later time points.

In the current analysis, we observed significant increases in serum sclerostin on day 90 and subsequent decreases on day 180 after administration of zoledronic acid. The early increase and subsequent decrease in sclerostin was consistent with the findings of other studies, but the timing was delayed. Although this difference may be partly explained by ethnic differences, there are no data measuring sclerostin at the same sampling point for comparison. Zoledronic acid decreases bone resorption by inhibiting osteoclasts at earlier time points, and the reduction in sclerostin at later time points may indicate the start of bone formation.

Correlations between parathyroid hormone (PTH) and changes in sclerostin levels have also been reported. Intermittent PTH has been shown to be associated with a reduction in sclerostin in rodents and in vitro studies [21, 22]. Moreover, serum levels of sclerostin are negatively correlated with the levels of serum PTH in patients with hyperparathyroidism [23]. Drake et al. investigated the change in sclerostin levels with administration of teriparatide 40 μg daily; significant decreases in serum sclerostin were observed during the 14-day treatment period [11]. Therefore, changes in endogenous PTH levels or treatment with teriparatide may contribute to changes in sclerostin levels. In our analysis, increases in iPTH at early time points were correlated with decreases in serum sclerostin after intermediate time points, suggesting that BP-related changes in iPTH may affect decreases in sclerostin.

It was also reported that another bone resorption inhibitor, denosumab, increased sclerostin levels at 6 months and maintained the levels thereafter [10]. On the other hand, no significant increase in PTH was observed after 3 months of treatment with denosumab [24], suggesting that the relationship between changes in levels of PTH and levels of sclerostin may differ depending on the type of bone resorption inhibitor used for treatment.

This study had several limitations. First, the correlation between iPTH and sclerostin was analyzed using combined data (4- and 5-mg groups), but the changes in serum parameters in each group indicated a similar direction and magnitude. Second, although a correlation between physical activity and serum sclerostin has been reported [25], the level of physical activity was not measured in this study.

In conclusion, once-yearly injection of zoledronic acid initially increased and subsequently decreased serum sclerostin through changes in iPTH levels; a decrease in sclerostin may indicate the start of bone formation during later time points after injection.

## Supplementary Information

Below is the link to the electronic supplementary material.

Supplementary file1 (PDF 144 kb)

## Data Availability

Not applicable.
