# Supplementary material for: Relationship Between Changes in Serum Levels of Intact Parathyroid Hormone and Sclerostin After a Single Dose of Zoledronic Acid: Results of a Phase 1 Pharmacokinetic Study
Source: Calcif Tissue Int. 2021 Aug 24;110(1):87–92. doi: 10.1007/s00223-021-00900-w (PMC8732852; doi:10.1007/s00223-021-00900-w)
Supplement: Supplementary file 1 — Supplementary file1 (PDF 144 kb) [file 223_2021_900_MOESM1_ESM.pdf]

Supplementary Information

Relationship Between Changes in Serum Levels of Intact Parathyroid Hormone and Sclerostin After a Single Dose of Zoledronic Acid: Results of a Phase 1 Pharmacokinetic Study

Journal: *Calcified Tissue International*

Authors: Tatsuhiko Kuroda[1] • Masataka Shiraki[2] • Toshitaka Nakamura[3] • Hiroaki Suzuki[4] • Kazuki Hiraishi[4] • Toshitsugu Sugimoto[5] • Satoshi Tanaka[6]

Affiliations:

[1]Healthcare R&D Center, Asahi Kasei Corporation, 1-1-2 Yurakucho, Chiyoda-ku, Tokyo 100-0006, Japan

[2]Research Institute and Practice for Involutional Diseases, 1610-1 Meisei, Misato, Azumino, Nagano 399-8101, Japan

[3]Touto Sangenjaya Rehabilitation Hospital, 1-24-3 Sangenjaya, Setagaya-ku, Tokyo 154-0024, Japan

[4]Medical Affairs Department, Asahi Kasei Pharma Corporation, 1-1-2 Yurakucho, Chiyoda-ku, Tokyo 100-0006, Japan

[5]Internal Medicine 1, Faculty of Medicine, Shimane University, 89-1 Enya-cho, Izumo, Shimane 693-8501, Japan

[6]Development Planning, Clinical Development Center, Asahi Kasei Pharma Corporation, 1-1-2 Yurakucho, Chiyoda-ku, Tokyo 100-0006, Japan

Corresponding author:

Satoshi Tanaka

Development Planning, Clinical Development Center, Asahi Kasei Pharma Corporation, 1-1-2 Yurakucho, Chiyoda-ku, Tokyo 100-0006, Japan

Tel: 81-03-6699-2376

E-mail: tanaka.sj@om.asahi-kasei.co.jp

Online Resource 1 Correlation of changes in calcium and phosphate with percent changes in iPTH using the whole set of data

		iPTH (days 0–15)		iPTH (days 0–29)	
		R	*p*-value	*R*	*p*-value
Serum calcium	Days 0–15	−0.673	< 0.001	−0.512	0.011
	Days 0–29	−0.424	0.039	−0.497	0.014
Serum phosphate	Days 0–15	−0.543	0.006	−0.197	0.356
	Days 0–29	−0.174	0.416	−0.287	0.174

iPTH intact parathyroid hormone

Online Resource 2 Correlation of percent changes in iPTH, calcium, and phosphate with percent changes in sclerostin using the whole set of data

		Serum sclerostin (days 0–90)		Serum sclerostin (days 0–180)	
		R	*p*-value	*R*	*p*-value
iPTH	Days 0–15	0.024	0.913	−0.214	0.314
	Days 0–29	0.018	0.933	−0.449	0.028
Serum calcium	Days 0–15	−0.013	0.952	0.260	0.220
	Days 0–29	−0.157	0.465	0.269	0.203
Serum phosphate	Days 0–15	0.059	0.785	0.045	0.834
	Days 0–29	−0.039	0.856	0.318	0.130

iPTH intact parathyroid hormone

Online Resource 3 Correlation between percent change in serum iPTH and sclerostin using the whole set of data

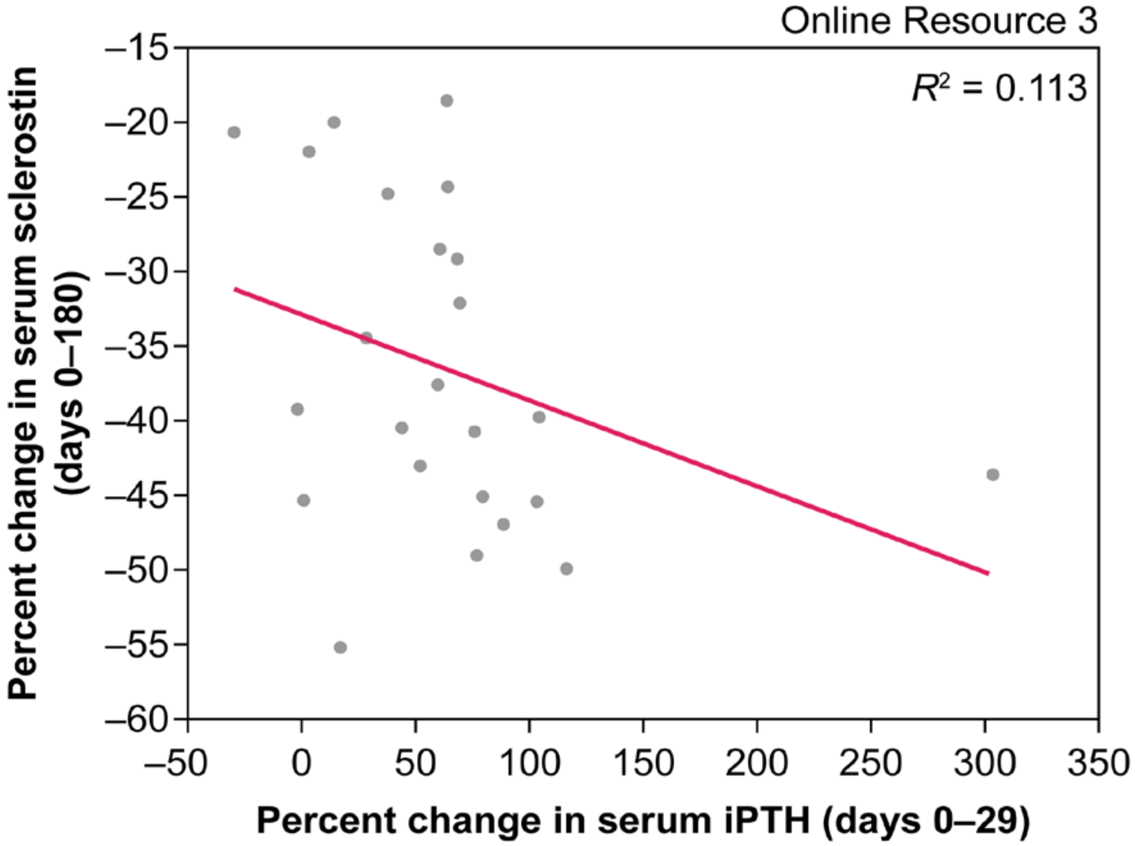

iPTH intact parathyroid hormone
